# The Crossroads of Periodontitis and Oral Squamous Cell Carcinoma: Immune Implications and Tumor Promoting Capacities

**DOI:** 10.3389/froh.2020.584705

**Published:** 2021-01-20

**Authors:** Omnia Elebyary, Abdelahhad Barbour, Noah Fine, Howard C. Tenenbaum, Michael Glogauer

**Affiliations:** ^1^Faculty of Dentistry, University of Toronto, Toronto, ON, Canada; ^2^Department of Dentistry, Centre for Advanced Dental Research and Care, Mount Sinai Hospital, Toronto, ON, Canada; ^3^Department of Dental Oncology, Maxillofacial and Ocular Prosthetics, Princess Margaret Cancer Centre, Toronto, ON, Canada

**Keywords:** periodontitis, oral pathogens, inflammation, oral squamos cell carcinoma, innate immunity, tumor microenvironment

## Abstract

Periodontitis (PD) is increasingly considered to interact with and promote a number of inflammatory diseases, including cancer. In the case of oral squamous cell carcinoma (OSCC) the local inflammatory response associated with PD is capable of triggering altered cellular events that can promote cancer cell invasion and proliferation of existing primary oral carcinomas as well as supporting the seeding of metastatic tumor cells into the gingival tissue giving rise to secondary tumors. Both the immune and stromal components of the periodontium exhibit phenotypic alterations and functional differences during PD that result in a microenvironment that favors cancer progression. The inflammatory milieu in PD is ideal for cancer cell seeding, migration, proliferation and immune escape. Understanding the interactions governing this attenuated anti-tumor immune response is vital to unveil unexplored preventive or therapeutic possibilities. Here we review the many commonalities between the oral-inflammatory microenvironment in PD and oral-inflammatory responses that are associated with OSCC progression, and how these conditions can act to promote and sustain the hallmarks of cancer.

## Periodontal Pathogens and the Host Responses: War and Truce

A strong correlative link exists between inflammation and the development and progression of many cancers. Approximately 20% of all human cancers have been related to chronic inflammatory conditions, raising speculation regarding the specific roles of inflammatory processes in driving carcinogenesis [1]. These highly-regulated multicellular responses involve a large network of both immune and stromal cells and result in major alterations in the abundance of reactive oxygen and nitrogen species, prostaglandins, cytokines, and chemokines within the microenvironment encompassing the cellular milieu in various tissues [2]. It can be inferred that initiation of tumourigenesis and tumor progression might be derived by these environmental modifications which can alter the cellular behavior and change the composition of the surrounding extracellular matrix.

In relation to the above, the putative role played by inflammation in tumourigenesis can be observed in the oral cavity [3]. The healthy oral cavity harbors an abundant commensal microbial community with varied microbial floral diversity. It represents one of the most ecologically complex niches within the human body where the oral microbiota–host equilibrium remains balanced in health conditions (truce) and shifts to disease state when immunoresponses are altered in retaliation to dysbiosis (war). Indeed, dysbiosis of the commensal oral microbiota and their subsequent invasion of the tooth supporting structures (e.g., the gingiva, periodontal ligament and bone) leads to the initiation and propagation of an inflammatory condition termed periodontitis or periodontal disease (PD) [4, 5].

**Figure 1:**
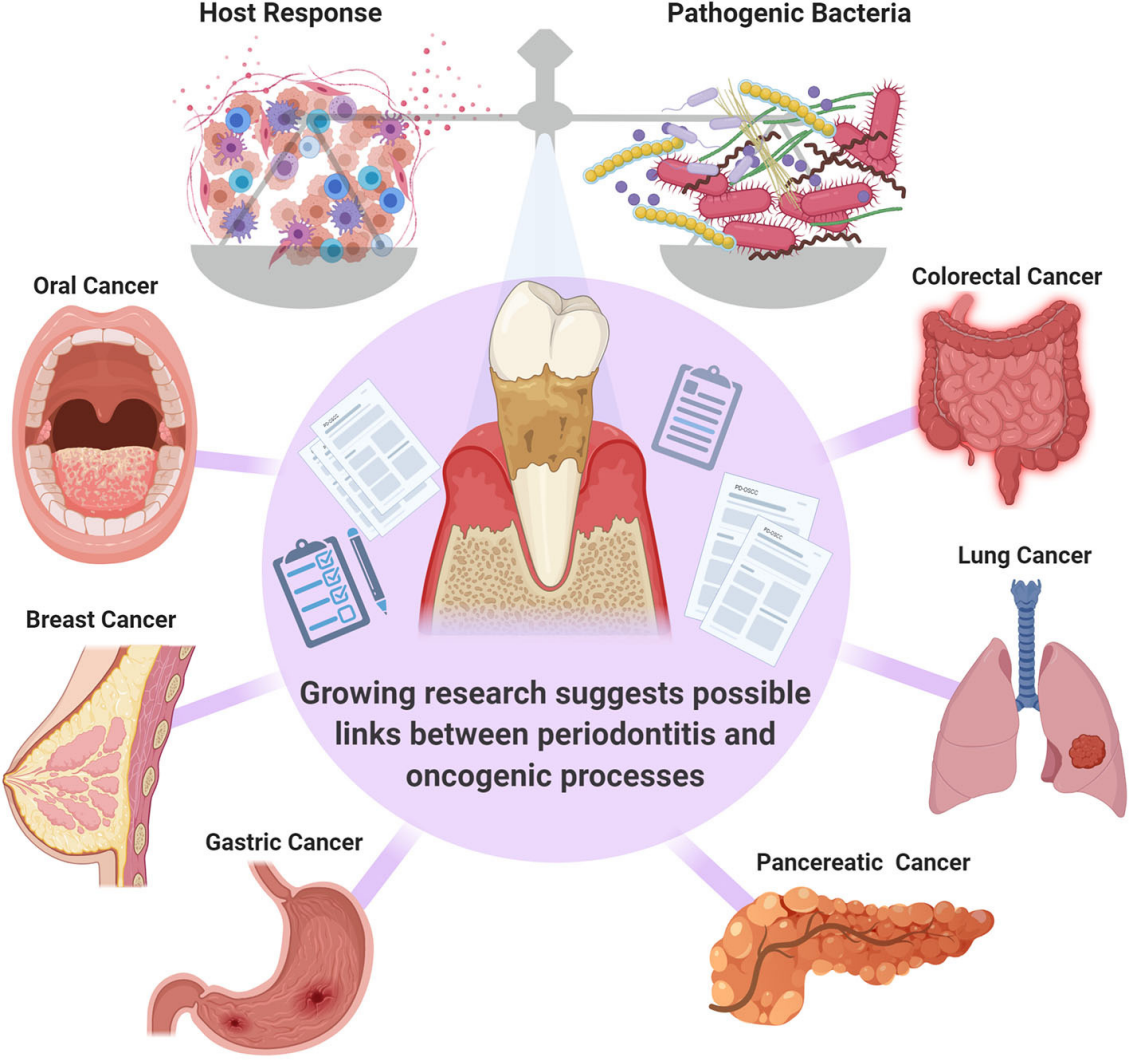
The balance between the pathogenic bacteria and host immune response is critical in governing the transition from health to disease. Systematic reviews suggest a link between periodontal inflammation oral [14], colorectal [11], breast [9], lung [12], gastric [13], and pancreatic [10] cancers. However, further confirmatory studies are required.

PD is considered to be, by far, the most common inflammatory condition affecting the oral cavity and it has been designated as an enabling characteristic of cancer development [6–8]. The presence of PD has been correlated to the presence of several types of malignancies including but not limited to breast, pancreatic and colorectal cancers [9–13] (Figure 1). However, whether this is a correlative or a causative association is not clear, and further investigation of the potential role of PD in cancer pathogenesis and potential systemic mechanistic effects is required. One group pointed out that although the link between PD with oral and other cancers has been well-documented in systematic reviews, these are often of limited power, and further research is necessary to fully confirm this association [8].

An association between PD and OSCC, two conditions that co-exist within the oral microenvironment, has been confirmed in many studies, suggesting that the immunologically distinct oral setting in PD could support tumor progression [14, 15]. Based on this observation one might reasonably predict that the most common site of primary squamous cell carcinomas in the oral cavity will be the gingival tissue, from which periodontal disease arises, however this was found not to be the case. Other sites in the oral cavity such as the lateral border of the tongue and the floor of the mouth have been recognized as common sites for OSCC and account for the majority of primary lesions [16]. This does not rule out the involvement of PD in supporting pathogenesis of OSCC at these sites. The specific sites where OSCC is commonly manifested in the mouth are known to have a greater permeability to carcinogenic factors compared to other oral tissues, and therefore are more susceptible to pathologenesis upon exposure to carcinogens [17]. The gingiva is covered by a thick protective layer of keratinized mucosa, which renders it less permeable to external carcinogenic insults, thus making it less affected [18]. Since the lateral border of the tongue and the floor of the mouth are common locations for premalignant oral conditions, the development of malignancies at these same areas may be a reflection of this pattern [19, 20]. Potential mechanisms through which the environmental conditions in the oral cavity associated with the presence of PD could support the progression of these lesions to develop carcinomas are the primary focus of this review.

Periodontal pathogens can contribute to carcinogenesis at locations other than the gingiva especially at the most common site, the lateral border of the tongue. Based on its anatomical location, this area comes in direct contact with the lingual aspect of mandibular molars, sites that have been reported to have the highest index of plaque accumulation and gingivitis [21]. Therefore, it can be inferred that the mucosa of the lateral border of the tongue is constantly exposed to the damaging effects of pathogenic periodontal bacteria and their toxins. Certain bacterial species, such as the putative periodontal pathogen *Porphyromonas. gingivalis* and *Tannerella Forsythia*, where detected in higher amounts in both the subgingival regions and the tongue of periodontally affected patients [22]. This supports the model that PD is a potential contributing factor or an accelerator to the development of primary oral cancer. Further studies are required to determine whether or not periodontitis can directly promote initiation of OSCC.

The gingiva has been identified as the most common site for metastatic carcinomas when compared to all the other soft tissues of the oral cavity [23]. These tumors have been reported as metastatic lesions of other primary cancers from distant sites including lung cancers, renal cell carcinomas, hepatocellular carcinomas and breast cancers [24]. Furthermore, metastatic gingival carcinomas have been strongly linked to the presence of teeth, which highlights the importance of the dentoalveolar structure in this pathogenic process [23]. Thus, the deregulated environment of gingival tissue in PD may form an attractive milieu for the seeding and growth of these metastatic cells.

A recent study suggested that periodontal pathogens can be regarded as a risk factor for PD independent of other well-known risk factors such as smoking, alcohol and human papilloma virus (HPV) infections [25]. Moreover, it has been suggested that periodontal therapy may result in a dramatic decrease in the risk of developing future OSCC [3]. This type of reversibility argues strongly in favor of the presence of a mechanistic link between PD and the development of both primary and secondary OSCCs. Given the high mortality and poor survival rate of OSCC (~50%), more investigations are being undertaken to determine what could be the underlying mechanisms that link PD with an increased risk of OSCC development [26]. In this review, we will explore the possible role played by the inflammatory environment created by PD in relation to carcinogenesis.

### Interaction of Malignant Cells and the Extracellular Matrix: Possible Links to the Development of Cellular Mutagenesis

When epithelial cells accumulate oncogenic mutations, complex interactions of these cells with their surrounding stroma contribute to initiation, progression and metastasis of OSCC [27]. It has been suggested that the deregulated microenvironment that exists in PD could contribute to various steps in carcinogenesis [28]. It has been well-established that chronic inflammatory conditions, including periodontitis, generate a mutagen-enriched environment [29]. Elevated levels of carcinogens such as reactive oxygen species (ROS) and bacterial-derived carcinogenic substances including volatile sulfur compounds, acetaldehyde, lactic acid, acetic acid, butyric acid, and isocaproic acid are associated with PD [30]. These compounds directly predispose the oral environment to promote host-cell DNA damage and the development of cellular abnormalities that may give rise to malignant transformation. The genotoxic effect of PD on buccal mucosa was assessed in an attempt to estimate whether or not PD can be used as a marker for genomic instability in oral tissues [31]. In accordance with the severity of PD, more DNA damage was observed in the form of nuclear bud formation, and chromosomal instability in cells of the buccal mucosa [31]. One of the most recognized genomic alterations in PD is that of the *TP53* gene encoding the p53 tumor suppressor protein, regarded as the *guardian of the genome*. When p53 is upregulated it has destructive effects that compromise periodontal integrity, suggesting its contribution to tumourigenesis [32]. Interestingly, the frequency of *TP53* gene expression in neoplastic conditions is similar to that seen in PD. Overexpression of *TP53* was linked to the pathogenesis of oral malignancies including squamous cell carcinoma and Kaposi's sarcoma, and was suggested to be a poor prognostic marker [33, 34]. Furthermore, the role of PD in cancer initiation has been demonstrated in a murine model where the size and number of OSCC lesions induced by the carcinogen, 4-nitroquinoline-1-oxide (4-NQO), were increased when putative periodontal pathogenic bacteria were co-introduced (orally) as compared to germ-free mice that received the carcinogen only [35].

In terms of the contribution of PD to cancer progression, it has been reported that a key periodontal pathobiont *P. gingivalis* is capable of disrupting immune surveillance by activating STAT3 signaling [36]. This in turn induces the generation of immunosuppressive myeloid-derived dendritic suppressor cells (MDSCs) from monocytes, which help to sustain oncogenic cell-proliferation and sponsor immune escape [36]. Periodontal-associated pathogens can also interfere with the expression of the Notch signaling pathway, the latter playing a major role in the development of chemoresistance in mutated cells [37].

#### Bidirectional Relationship Between OSCC and PD

Recent findings suggest that the link between PD and OSCC could be reciprocal or bidirectional, with OSCC contributing to the development and/or the exacerbation of PD and *vice versa*. When the carcinogen 4-NQO was administrated to rats in order to induce the development of OSCC, the group of rats that developed OSCCs upon 4-NQO exposure had significant spontaneous alveolar bone resorption compared to both the control group that did not receive 4-NQO and the treated-group that did not develop OSCC. Thus, this enhanced alveolar bone loss could be correlated to the development of squamous cell carcinoma lesions in the oral cavity [38]. Interestingly, Pushalkar et al. had found significant variations in the microbiota diversity between tumor and non-tumor sites in individuals affected with OSCC [39]. In that study, tumor sites tended to have more distinct pathogenic microbial populations than those found at non-tumor sites in the same subjects. This microbial shift was suggested to induce a chronic inflammatory state at tumor sites, which can support the progression of adjacent tumors. This highlights the interconnectivity of both diseases, and how changes in the oral environment could induce conditions that are permissive to the development of either PD, OSCC or both.

Although the inflammatory oral microenvironment has been recognized to orchestrate a predominantly pro-tumor immune response, there are also paradoxical effects that can be anticarcinogenic one of which is that of the CXCL14 chemokine [40]. CXCL14 is normally expressed in healthy oral tissues yet it is decreased or absent in malignant oral epithelium [41]. CXCL14 was found to completely block OSCC establishment in SCID mouse xenografts [42]. A more recent study, explained that the anti-tumor functions exerted by CXCL14 are carried out by means of supressing PD-L1 expression and NF-*k*B mediated EMT [43]. Periodontal pathogen *P. gingivalis* is able to stimulate the expression of the CXCL14 from oral epithelial cells both directly and by antagonizing epidermal growth factor (EGF)-induced activation of the MEK-ERK1/2 pathway known to repress CXCL14 transcription [44]. This CXCL14 involvement in PD may be considered as one of the methods by which anti-tumor responses are supported. However, further research will be necessary to understand the complex interactions between PD and OSCC.

## OSCC Immune Permissive Environment Induced by Periodontal Disease

**Figure 2:**
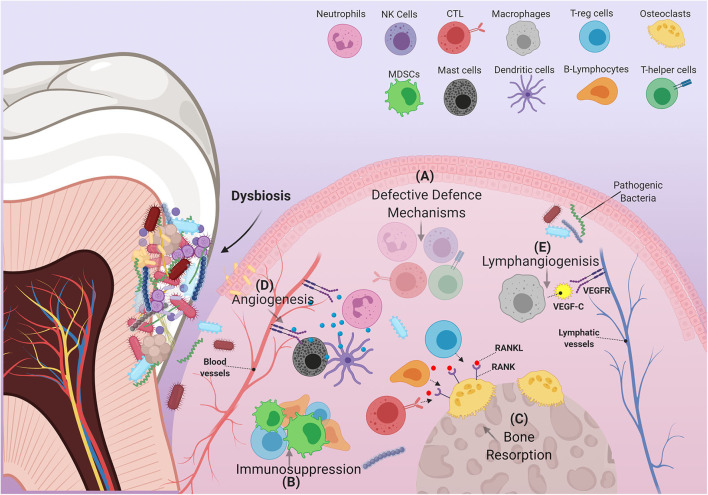
Schematic illustration of immune cells in inflamed periodontium. The figure shows how the immune cell infiltrate in PD can exert several alterations within the gingival tissue environment that are able to support the pathogenicity of OSCC. **(A)** In PD, many immune cells fail to carry out some of their characteristic defensive functions. These cells include, but are not limited to, (1) Neutrophils: decreased defensins and defective NETs formation, (2) cytotoxic T-lymphocytes: decreased IFN-γ production, (3) T-helper cells: suppressed effector Th1 response, and (4) natural killer cells: reduced granzyme and IFN-γ production. **(B)** Immune suppressive cells: MDSCs, T-regs, B-regs (a subtype of B-lymphocytes) are recruited to the site of inflammation, disrupting immune surveillance. **(C)** Increased RANKL expression by several immune components including T-regs, B-lymphocytes and Cytotoxic T-lymphocytes, induces resorption of alveolar bone. **(D)** Expansion of the vascular compartment of the periodontium is mediated by several factors produced by certain immune cells. These include MMP-9, which is mainly produced by neutrophils and dendritic cells, as well as many angiogenic products released by mast cells (VEGF, tryptase, heparin, histamine, IL-8, basic fibroblast growth factor). **(E)** Macrophages contribute to the expansion of lymphatic vessels by producing VEGF-C.

Intense focal influx of immune cells into sites affected by pathogenic bacteria leads to a cumulative build-up of their numbers within the oral environment [45]. Changes in the behavior of immune cells under the critical influence of pathogenic bacteria, or interactions between these cells and other immune-regulatory networks e.g., cytokines, chemokines, and growth factors can disrupt productive immune surveillance in PD [46]. Any “corruption” of the immune response could hypothetically permit the homing of metastatic tumor cells into these sites or possibly induce the development of primary malignant lesions. Interestingly, in PD many immune cells adopt behaviors or characteristics similar to those observed in immune cells recruited by tumors that favor their progression. Hence, the “re-education” of dysfunctional oral immune cells by means of periodontal therapy could conceivably lead to beneficial results insofar as the development and/or treatment of OSCC are concerned. Herein, we discuss how cells of both the myeloid and lymphoid lineages are affected by PD, and how they contribute to the etiopathology of OSCC (Figure 2).

### Myeloid Derived Cells

#### PMNs

Neutrophils, also called polymorphonuclear leukocytes (PMNs), are the most abundant primary innate immune responders in the gingival crevice and periodontal pocket. They play an indispensable role in maintaining periodontal health and their greatly upregulated recruitment is observed in response to oral microbial dysbiosis [47–50]. PMNs have been implicated in prolonging the extent and severity of inflammatory PD leading to substantial damage to bone and soft connective tissues surrounding the teeth [51, 52]. In cancer, different PMN subsets with either anti-tumor or pro-tumor characteristics have been identified. This has led to the emergence of a new classification of PMNs, based on anti-tumor (N1) and pro-tumor (N2) cancer-associated PMNs [53]. In OSCC, it was noted that an increase in the oral PMN infiltrate is correlated with a reduced survival rate and an increased recurrence rate [54]. Furthermore, co-cultures of peripheral blood PMNs and OSCC cells were found to increase the invasiveness of the cancer cells, independently of direct cell contact [55]. Hence, the presence of abundant pro-inflammatory PMNs in PD could contribute to the pathogenesis of oral cancers.

In PD, changes in oral PMN phenotype were observed. This was characterized by PMNs that survived longer in the oral cavity due to upregulation of anti-apoptotic genes of the Bcl-2 family, and downregulation of pro-apoptotic transcripts [56]. Moreover, direct interaction between PMNs and regulatory T-cells (T-regs) that had been stimulated with lipopolysaccharides (LPS) promoted an abnormal production of the immunosuppressive cytokine IL-10 by the PMNs, and these IL-10-producing PMNs were observed in the oral cavity of patients with PD [57]. Interestingly, similar, longer lived, IL-10+ PMN phenotypes were identified in the tumor microenvironment (TME) of OSCC [58, 59]. These PMNs displayed a clear upregulation of the anti-apoptotic genes of both the Bcl-2 and NF-*k*B families upon entering the TME and transitioning into N2 tumor associated neutrophils (TAN).

Another common feature between PMNs associated with both OSCC and PD is the downregulated expression of PMN gelatinase-associated lipocalin (NGAL) [60, 61]. The downregulation of NGAL observed in OSCC has been implicated in improving the survival of cancer cells while upregulating their ability to migrate and invade tissues. This is related to the activation of mTOR signaling and the suppression of autophagy as a result of reduced expression of LC3B [61]. The resemblance between PMN behavior in the microenvironment of both PD and OSCC, suggests that PMNs in PD can augment and potentiate the development of OSCC by exerting the same pro-tumor functions as TANs in the oral microenvironment.

PD deregulates the expression of many PMN-secreted products, which can impair certain PMN functions and provide a favorable environment for cancer cells to thrive. For instance, the defensins, human neutrophil peptides 1, 2 and 3 (HNP1-3), are known to induce cytolytic effects on their target cells, particularly cancer cells. It has been suggested that higher levels of defensins might promote preferential oncolysis and provide a better host response to tumor invasion (i.e., prevention). Suppression of antimicrobial peptides HNP1-3 was observed in patients with PD [62]. This suppression could therefore be anticipated to cause a loss of PMN mediated oncolysis. Additionally, in PD, PMN-mediated expression of pro-angiogenic matrix metalloproteinase-9 (MMP-9) is accompanied by a substantial decrease in the expression of their inhibitors, tissue inhibitors of matrix metalloproteinases (TIMPs) [63, 64]. In combination, this unopposed upregulated MMP-9 expression can lead to conditions that are permissive for angiogenesis required for tumor growth and intravasation and therefore, progression of tumors such as OSCC.

In chronically inflamed gingival tissue, increased PMN-expression of a proliferation-inducing ligand, known as APRIL, was shown to increase the proliferation, survival, and invasion capacity of tumor cells in oral cancer [65]. An increased rate of tumor growth and aggressiveness was associated with APRIL-rich breast cancers compared to those with reduced expression of APRIL [66]. Other PMN products such as neutrophil elastase (NE), which have the capacity to degrade most extracellular proteins, has been implicated as a biomarker in both PD as well as head and neck squamous cell carcinoma (HNSCC). A direct link between increased levels of NE in PD and its potential role as a co-factor in the initiation and advancement of OSCC has recently been established. A considerable increase in salivary levels of NE in patients with combined PD and OSCC, as compared to those affected by either PD or OSCC, highlights the important role of NE in contribution to oral cancer [67].

One of the strategies that PMNs use to counteract infectious agents is formation of web-like neutrophil extracellular traps (NETs) [68]. These extruded structures, generated by a process referred to as NETosis, are composed of chromatin and are able to trap pathogenic moieties and destroy them by delivering high concentrations of anti-microbial agents such as histones, proteases and PMN granule proteins [69]. Excessive NETosis, evident in exacerbated inflammatory conditions, has been linked to unfavorable consequences such as autoimmune diseases, thrombus formation and cancer metastasis [70]. In HNSCC, it has been proposed that monitoring NET levels can be a successful approach to predict if patients are at a high risk of developing metastatic tumors [71]. PMN NETs are highly prevalent in PD, and may therefore stimulate the metastatic potential of oral cancer cells [49, 72]. Furthermore, NETs formed under the influence of PD were found to have defective functions. *P. gingivalis*, which can cleave the protease-activated receptor-2 (PAR2) on the surface of PMNs, was found to trigger excessive formation of NETs that lacked their characteristic bactericidal activities [73]. These NETs were unable to restrain and kill *P. gingivalis* and other pathogenic bacterial species, allowing them to thrive. Although it hasn't been extensively explored, it can be suggested that this defective function of NETs can also be beneficial to cancer cells, allowing them to escape PMN-mediated destruction.

Collectively, these findings indicate that PD is associated with functional alterations in the oral PMN population, which suppress PMN immune surveillance by modifying the production of many PMN-secreted products. These changes are not dissimilar to characteristics of the N2 pro-tumor PMN. Accordingly, in the presence of PD, the PMNs that are recruited to the OSCC TME could augment the progression of cancer instead of limiting its progression.

#### Macrophages

In addition to PMNs, macrophages play a central role in the immune response observed in PD and contribute to both the onset and resolution of inflammation [74]. Functionally, macrophages can be classified into two main phenotypes, M1 and M2, based on the type of activation they acquire upon interacting with the microenvironment at diseased sites. M1, or classically activated macrophages, differentiate in the presence of IFN-γ, LPS, or TNF-α; and are the primary producers of pro-inflammatory cytokines, such as IL-1, IL-12, and TNF-α [75]. Additionally, these cells have potent anti-tumor capabilities and treatments that can induce tumor-associated macrophages to adopt an M1 phenotype hold potential therapeutic promise [76]. M2, or alternatively activated macrophages, are induced in the presence of IL-4, IL-10, or IL-13 [74]. They are involved in immunoregulation, tissue repair, and angiogenesis, and are linked to carcinogenesis and tumor progression [77].

Although PD is more often associated with elevated numbers of M1 anti-tumor macrophages [75, 78], this does not necessarily rule out the presence of M2 pro-tumor macrophages. In a recent study, the effect of periodontal inflammation on macrophages demonstrated the presence of increased numbers of M1 macrophages as well as elevated numbers of M2 macrophages [79]. Furthermore, when screening for changes in cytokine expression in gingival tissue derived from participants with PD, the M1 cytokines, TNF-α and IL-1β, and the M2 cytokine, IL-10, were all significantly increased [79]. These findings have challenged the prevalent concept of macrophages being solely pro-inflammatory within the context of PD, and have proposed the presence of a more heterogeneous macrophage population in PD. Based on this, it cannot be assumed that changes in macrophages in the presence of PD will only favor the pro-tumor response.

Secreted factors produced by macrophages can play a role in the pathogenesis of OSCC. For instance, elevated levels of TGF-β1 in the PD environment can induce vascular endothelial growth factor-C (VEGF-C) production by macrophages, which stimulates the proliferation of new lymphatic vessels through stimulation of VEGF receptor-3 (VEGFR-3) on lymphatic endothelial cells [80]. In the same context, inflammatory macrophages had been recognized previously to be able to transdifferentiate into lymphatic endothelial cells that express endothelial markers such as LYVE-1 and Prox-1, thereby integrating into lymphatic vessels promoting their expansion [81]. In cancer, this can have a negative impact on the overall prognosis for patients with OSCC, as increases in microvascular density might support metastasis of malignant cells to nearby and distant lymph nodes. This was shown when OSCC of the tongue was studied. In this regard, higher levels of VEGF-C were considered to predict poor cancer-specific survival [82].

Macrophage migration inhibitory factor (MIF) has been noted to play a role in both periodontitis and cancer. MIF is a well-known pro-inflammatory effector cytokine expressed by a variety of immune and non-immune cells including macrophages. In inflammation, it promotes the migration and recruitment of leukocytes to the infected sites in response to pathogenic stimuli. Investigations have recognized MIF as a central participant in the cancer-associated immune response, having the capacity to support the development of HNSCC by its pleiotropic roles in mediating hypoxia response, angiogenesis, and epithelial-mesenchymal transition (EMT) [83]. Moreover, gene inactivation of MIF receptor (CD74) or the use of MIF agonist ISO-1 resulted in a significant reduction in the proliferation of gastric cancer cells [84]. Therefore, the higher concentrations of serum and salivary MIF observed in PD might stimulate the proliferation, metastasis and invasion, of oral cancer cells into other tissues [85].

#### Myeloid-Derived Suppressor Cells

The expansion of immune suppressive cells, including myeloid-derived suppressor cells (MDSCs), and their accumulation at the sites of malignant lesions is one of the main mediators of cancer progression [86]. Not only do these cells contribute significantly to the immune response regulation, but they also provide an environment that is capable of sustaining angiogenesis, stemness, and metastasis [86]. Although these recently discovered cells have not been extensively studied in the context of PD, emerging evidence suggests that they are more involved in PD and in PD induced OSCC than previously suggested. Strains of *P. gingivalis* that express Mfa1-fimbriae were found to mediate immune escape by invading monocytes and inducing their differentiation into myeloid-derived dendritic suppressor cells (MDDSCs), which were characterized by being highly resistant to apoptosis [36]. Additionally, these cells appeared to be compatible with the immune suppressive environment created by the indoleamine 2, 3 dioxygenase (IDO) enzyme found at chronically inflamed sites including PD.

Furthermore, the expansion in the MDSC population has been linked directly to promoting the development of either primary or metastatic cancers in the oral cavity as follows. A recent study revealed that *P. gingivalis* can induce differentiation of MDSCs from monocytes, and also invade oral pre-malignant lesions thereby inducing a substantial influx of MDSCs [87]. This is achieved by upregulating the expression levels of certain chemokines (CCL2 and CXCL2) and cytokines (IL-6 and IL-8) in dysplastic oral keratinocytes. Accordingly, this “biases” the immune system toward supporting oncogenesis. Hence, this suggests that patients with PD could be more susceptible to developing primary oral cancers from pre-existing oral dysplastic lesions.

It has been suggested that periodontal inflammation can shape the pre-metastatic immune microenvironment in the gingiva. In a very recent study, gingival fibroblasts showed increased production of IL-1β upon sustained exposure to LPS, which resulted in increased expression of CCL5, CXCL12, CCL2, and CXCL5, potent chemotactic factors for MDSCs [88]. This generates a pre-metastatic niche at sites of inflammation, which promotes metastatic events, for example, progression of breast cancer to head and neck tissues [88].

#### Dendritic Cells

Dendritic cells (DCs) are specialized antigen presenting cells (APCs) that can be activated by different stimuli including bacteria, viruses, and damaged tissues, thereby potentiating their phagocytic activities [89]. These cells have been linked strongly to the induction of T cell responses and therefore it has been suggested that they play a key role in mediating anti-tumor immunity [90]. However, compelling evidence indicates that the function of DC is impaired in cancer. Decreased function of DC is thought to be related to the presence of immunosuppressive conditions that induce a network of factors capable of suppressing these cells [91]. A greater understanding of how DCs respond to PD is necessary in order to determine how this can contribute to tumor immune responses.

Periodontal pathogens can, in many ways, alter the response of DCs to suppress the local immune surveillance mechanism, allowing them to invade periodontal tissues. For example, LPS derived from *P. gingivalis* has been shown to induce alterations in the MMP-9/TIMP-1 ratio in DCs similar to those described previously for PMNs. MMP-9 is expressed in significant amounts in concert with reduced production of TIMP-1 resulting in a higher MMP-9/TIMP-1 ratio that favors local tissue destruction. In laryngeal cancer, higher levels of MMP-9 were correlated with increased counts of tolerogenic DCs [92]. These tolerogenic DCs have altered functionality and produce immune-regulatory molecules, including retinoic acid and TGF-β, so that they appear to induce the development of laryngeal cancer specific T-regs that suppress the production of the cytotoxic T-lymphocyte effector molecule granzyme B [93]. Moreover, it was found that *P. gingivalis* strains having minor fimbria can bind to the DC-SIGN receptor expressed by DCs [94]. This binding is followed by internalization of these bacterial strains which was shown to alter DC function, retarding their maturation and minimizing their production of inflammatory cytokines. This also results in a Th2-biased weak immune response that promotes immune escape [94].

Plasmacytoid dendritic cells (pDCs) are a unique subset of DCs that can detect the nucleic acids of pathogenic microorganisms and respond rapidly by releasing an enormous amount of type 1 interferon (INF) [95]. The negative regulation of these cells by TAM signaling was found to be repressed in PD, which explains their increased influx in the gingiva of patients with PD [96]. Later studies have indicated that the number of tumor-infiltrating pDCs is linked with lymph node metastasis and poorer prognostic outcomes in patients with OSCC as these cells were dysfunctional with relatively low production of IFN-α, IL-6, and TNF-α as compared to circulating pDCs [97]. This functional impairment came as a result of many inhibitory factors present in the TME including VEGF, TGF-β, and IL-10 which inhibit the activation of pDCs and prevent antigen presentation. Therefore, although PD appears to induce higher pDC recruitment in oral cancer, these cells are functionally repressed by the TME, and therefore fail to contribute to anti-tumor immunity. Therefore, current results support the model that PD suppresses anti-tumor functions of DCs, indicating that reduction in periodontal inflammation could restore anti-tumor functions of DCs.

#### Mast Cells

Mast cells (MCs) are immune cells linked principally to allergic reactions, but they are also functionally associated with many other pathological conditions including cancer and PD [98, 99]. Morphological features include their distinct cytoplasmic granularity related to the presence of unique basophilic granules that are rich in inflammatory mediators including histamine and heparin [100]. These cells reside in all the connective tissue components of the oral cavity, including the periodontal ligament and gingiva, and are distributed mainly along the available vasculature [101]. Their unique location, in close proximity to other immune responders, allows them to rapidly take action whenever they sense changes within their immediate microenvironment. In PD, constant irritation from the plaque biofilm stimulates MCs to proliferate, which denatures the composition of the normal gingival tissue [102]. This increase in number results in differential expression of MC mediators within the oral connective tissues, and the subsequent functional consequences of this have the potential to contribute directly to the pathologic processes of OSCC.

Other than the basophilic granular content of MCs, they have the ability to synthesize and express many mediators that induce several characteristic functions; such as the chymase and tryptase proteolytic enzymes. In PD, the production of MC chymase has the ability to activate many factors including collagenase, MMP-3, and MMP-9 which are known to induce disruption of the basement membrane [102, 103]. As such, the increase in MC chymase levels can reflect one potential means by which MCs promote the progression of oral malignant and premalignant lesions. Although it has not been studied in OSCC, the increase in levels of chymase has been shown to influence prostate cancer progression via downregulation of expression of signals for the androgen receptor expressed in prostate cancer cells thereby increasing the invasive ability of these cells [104]. As for tryptase, the severity of periodontal inflammation was found to be correlated positively with its expression levels [105]. This elevated expression can impact the role MCs play in cancer as MC-derived tryptase is considered a key fibroblast activator prompting them to adopt a tumor-favoring phenotype [103]. MC-activated cancer-associated fibroblasts (CAF) stimulate the migration, proliferation, and malignant transformation of keratinocytes and have the ability to alter the tumor immune response by their sustained TGF-β production, which regulates the activity of natural killer (NK) cells [106].

Another function of MCs, which might be linked to their role in evolving malignant lesions, is their ability to prompt MC-directed vascular expansion. Given their strategic location in close proximity to the oral vasculature, they are able to drive neovascularization aided by angiogenic factors, including VEGF, or substances that can have angiogenic properties, such as tryptase, IL-8, TNF, basic fibroblast growth factor (bFGF), heparin, and histamine [101]. Angiogenesis facilitates the invasion and metastasis of cancer cells by remodeling the TME, and provides increased oxygen and nutrients for tumor growth. In the context of OSCC, a positive correlation between the MC and vascular densities in OSCC has been recently established, suggesting the ability of these cells to regulate angiogenesis [107]. This may explain the aggressive behavior and metastatic tendencies of these oral tumors. Although the angiogenic effects of MCs are only one of many pathways that induce vascular expansion within the TME, which responds to angiogenic factors from a variety of different sources, it is clear that MCs are effective inducers of this process and their role should be further investigated.

### Lymphoid Derived Cells

#### Regulatory T-Lymphocytes

In PD, when dysbiotic bacteria attack the gingival tissue, an exaggerated host immune response is triggered in an attempt to avoid the dissemination of these micro-organisms, which results in tissue damage [108]. Consequently, Immunosuppressive T-regulatory cells (T-regs) are recruited into these sites to attenuate this aberrant inflammatory response and promote immune homeostasis. The main purpose of these cells is to minimize collateral tissue damage, which in turn promotes the survival of periodontal pathogens [109]. These cells limit the immune response by modulating the activities of other immune cells by a number of mechanisms, including: (1) binding of the T-reg expressed CTLA-4 to CD80/86 on DCs which inhibits co-stimulatory signals; (2) limiting the availability of IL-2 needed for effector T-cell activation; (3) producing immunosuppressive factors (ex. TGF-β, IL-10, IL-35); and (4) IDO-mediated T-cell dysfunction [110]. As one of the central players in PD, the recruitment of these immunosuppressive cells into the oral milieu will impact the development of further diseases including malignancies. Indeed, infiltration of Foxp3+ T-regs into the oral environment was shown to promote malignant transformation of oral premalignant lesions giving rise to OSCC [77].

It has been noted that CCL20, a chemokine linked with recruiting CCR6-expressing cells (including CCR6+ T-regs), is upregulated in both gingival fibroblasts and in periodontal ligament cells during periodontal inflammation [111, 112]. Co-stimulation of gingival fibroblasts by IL-1β and IL-22, present in the inflammatory milieu of PD, enhanced the activation of the C-Jun N-terminal kinase and the NF-κB pathway resulting in the upregulation of CCL20 [111]. In a similar manner, IL-6/sIL-6R stimulation enhanced CCL20 production in IL-1β-stimulated periodontal ligament cells by activating the STAT3 signaling pathway [112]. Although CCL20 has been mainly linked to recruiting Th17 cell, a correlation between the expression of CCL20 and FOXP3 mRNA in OSCC was noted. This led to a further study demonstrating that oral cancers with high CCL20 expression favor the selective recruitment of CCR6+ T-regs [113]. These CCR6+ cells are phenotypically and functionally distinct from CCR6- T-regs and possess enhanced suppressive activity, IL-10 production and FOXP3 expression [113]. Further studies are necessary to determine if the intensified recruitment of CCR6+ T-regs by CCL20/CCR6 signaling in PD can promote the progression OSCC.

In PD, the triad of molecules that regulate bone hemeostasis/pathology, receptor activator of nuclear factor-κB ligand (RANKL), its receptor RANK and its decoy receptor osteoprotegerin (OPG) are imbalanced resulting in bone loss. RANKL, which is upregulated in PD, promotes T-reg induction, while RANKL inhibition interferes with the migration of T-regs, and induces lower expression levels of the T-reg-related cytokines IL-10 and TGF-β [114]. This RANKL-mediated T-reg activation is further sustained in PD by the downregulation of osteoprotegerin that can block RANKL in the saliva and GCF of patients with PD [115, 116]. This RANKL–RANK signaling in OSCC has been shown to stimulate cancer metastasis in concert with the presence of active tumor-infiltrating T-regs [117]. Therefore, the imbalance of the RANKL/RANK/OPG signaling in PD may contribute to developing a T-reg mediated downregulated immune response which allows oral cancer cells to evade immune destruction.

#### Natural Killer Cells

Natural killer cells (NKs) are innate lymphoid cells that are actively involved in the destruction of foreign, infected and cancerous cells that are deemed to be of danger to the host [118]. Their cytotoxic activity is mediated by trafficking their granular content into the cytosol of their targets resulting in their lysis in a granzyme B and perforin-dependent fashion. Additionally, these cells produce an array of pro-inflammatory cytokines and chemokines (IFN-γ, TNF, IL-6, GM-CSF, and CCL5) that shape both the innate and adaptive immune response [119]. In PD however, the NK population has been linked to unresolved immune responses leading to the progression of PD [120]. This is due to immunosuppressive molecules in the periodontal inflammatory microenvironment that are capable of suppressing these NK cells.

Immune suppressive molecules including (IL-10, TGF-β, and IL-35) were found to be expressed locally as well as systemically under the impact of PD [121, 122]. In OSCC patients, IL-10 and TGF-β have been found to reduce the ability of peripheral NK cells to lyse their targets, and reduced their expression of IFN-γ, their key effector molecule [123]. TGF-β specifically has been found to severely compromise an important stimulator of the effector function of cytotoxic lymphocytes and NKs. NKG2D, a receptor found on the surface of these cytotoxic cells, upon engaging with their NKG2D ligand expressed on tumor cells a stimulatory signal is transmitted into these cells allowing immunosurveillance of cancer cells [124]. TGF-β was found to interfere with this process by reducing the NKG2DL expression by tumor cells and downregulating NKG2D on NK cells [125]. On the other hand, IL-35 has the ability to reduce granzyme B production in NK cells impeding this cytolytic defense mechanism [126]. Another cytokine readily available in PD, that may contribute to impairing NK function, is IL-17 which plays a role in accelerating the phosphorylation of GSK-3β [127]. This increased GSK-3β phosphorylation was found to be involved in weakening the cytotoxic response of NK cells. These findings highlight several NK suppressive mechanisms adopted in PD that can compromise the effectiveness of the NK mediated anti-tumor immune response.

Pathogenic microorganisms might also contribute to modulating the activities of NKs. This was observed recently when *Fusobacterium nucleatum*, a pathogen strongly associated with PD, was discovered to bind to CEACAM1 (an inhibitory receptor on various immune cell subsets) [128]. The activation of CEACAM1 impairs degranulation of NK cells preventing the release of their lytic content and also diminishes their production of the immune-activating cytokine IFN-γ. Therefore, due to the suppressive influence of cytokines and microorganisms in PD, it is most likely that these cells will not function efficiently to kill tumor cells in OSCC.

#### Cytotoxic T-Lymphocytes

Cytotoxic T-lymphocytes (CTL) are the key effector arm of the adaptive immune response. They adopt one of three mechanism to mediate the clearance of foreign, infective or malignant cells: (1) releasing cytokines that stimulate killing of their targets, predominantly TNF-α and IFN-γ; (2) binding of their Fas ligand of to the Fas receptor expressed on their targets, triggering apoptosis; and (3) trafficking pre-synthesized destructive granzymes and perforin into the intercellular space of the opposed cells [129]. Increased levels of CTL were observed in PD, yet, these cells were in fact linked to the etiopathogenesis of PD, leading to a more rapid onset of severe tissue destruction [129].

Recent studies postulate that the different cellular and molecular elements found within the inflammatory setting of PD may modify the function of CTLs. For example, the increased frequency of MDSCs, as described earlier in PD, has been confirmed to impede the function of CTLs in OSCC [130]. These MDSCs hamper expression of the CD3-ζ chain on CTLs, modifying the cytoplasmic signaling that leads to their activation [130]. MDSCs have also been implicated in decreasing IFN-γ production from these cytotoxic cells, impairing one of their most prominent destructive mechanisms [130]. Finally, MDSCs suppress the proliferative ability of CTLs, through inhibiting the TCR-driven cycling [130]. Together, this supports the establishment of an inflammatory state that supports the advancement of tumors.

Another mean by which PD can limit functionality of CTLs is through the production of programmed death-ligand 1 (PD-L1), a ligand that signals for T cell apoptosis. The binding of PD-L1, a transmembrane protein belonging to the B7 family, to its receptor (PD-1) is considered an important factor for suppression of the adaptive immune response. It can result in stunting the activities of immune cells leading to their apoptosis. The upregulated expression of both PD-1 and PD-L1 on CTLs, with the concomitant increase of PD-L1 mRNA in saliva of PD patients will, without doubt, affect the immune response in OSCC [131, 132]. The effect of these CD8+PD-1+ cells was observed in OSCC, where they were found to be less responsive than those CD8+ cells lacking PD-1 expression [133]. This is consistent with the observation that higher grades of oral premalignant and malignant lesions were positively linked to higher expression of PD-1/PD-L1 [134].

#### B-Lymphocytes

B cells are regarded for their role in mediating the humoral part of the adaptive immune response given their chief role in antibody production [135]. They have been subdivided into many different subsets depending on various contacts and cytokine stimuli present within the local issue in which they respond. One of these functional B cell subsets, which is significantly increased in PD is the IL-10 producing regulatory B cells (B-regs) [136]. The conversion of human B cells into B-regs was found to be induced by the immunosuppressive cytokine IL-35, which is, as previously mentioned, upregulated in patients with PD [122, 137]. These recruited cells halt the progressive bone loss associated with PD by modifying the expression of pro-inflammatory cytokines and local proliferation of Th17 cells [136]. The immunosuppressive properties of these cells are likely to impact oral malignancies, for example, in tongue squamous cell carcinoma, the frequency of B-regs within the microenvironment was positively correlated to IL-10 mediated conversion of CD4+T cells to T-regs and thus was an indicator of poor prognosis [138].

One of the most notable features of B cells that were observed in both OSCC and PD was their distribution. In these diseases B cells were mainly arranged on the periphery and peri-lesion areas mostly toward its advancing edge [139, 140]. Furthermore, in PD all B cell subsets had elevated expression of RANKL, promoting osteoclast formation and bone recession [141]. In a parallel phenomenon, B cells promoted invasion and metastasis of oral cancer cells, and increased B cell infiltration in OSCC was significantly correlated with lymph node metastasis [140].

From another perspective, growing evidence supports the involvement of Epstein-Barr virus (EBV) in the pathogenesis of PD. This is linked directly to the function of B cells present in the PD inflammatory microenvironment, as EBV infects B cells promoting their expansion and differentiation before remaining latent in the circulating memory B cells [142]. Through this, PD supports the amplification and viral spreading of EBV in a B cell dependent manner. This may favor the development of OSCC, as the vast majority of OSCC cases are located in B cell rich areas, with EBV+ tumors having a more rapid onset and more invasive potential [143].

Although the role of B cells in both PD and OSCC has been understudied, the findings summarized here are promising, and suggest an important role of these cells.

#### T-Helper Cells

T-helper (Th) cells are pivotal moderators of the inflammatory immune response. They assist the other leukocytes in delivering an effective immune response by several means such as: the induction of B-cell maturation; CTL and macrophage activation; and recruitment of PMNs [144]. Several subsets of these CD4+ T-lymphocytes have been characterized including Th1, Th2, Th3, Th17, and T follicular helper (Tfh) cells, with each harboring unique features and functions [145]. Originally, Th1 and Th2 were shown to play a role in the pathogenesis of PD. However, with the emergence of new Th subsets and a deeper understanding of their plasticity under the influence of environmental conditions, this Th1/Th2 model has been altered considerably [146]. Herein, we discuss how the effects of each of the three dominant Th subsets in PD (Th1, Th2, & Th17) might also contribute to OSCC pathogenesis.

Th1 cells functions by enhancing phagocytosis, complement fixation, and opsonization, strengthening the immune response against pathogens. Th1 cells differentiate from naïve T cells under the influence of the inductive signals IL-12 and IFN-α. In PD, an upregulation of GATA-3 signaling was noted [147]. GATA-3 was previously reported to suppress STAT4 signaling and thereby limit the differentiation capacity of Th1 cells and in the meantime enhance that of Th2 cells [148]. Likewise, overexpression of GATA-3 was identified in OSCC samples, consistent with another study reporting that Th1 cytokines (IL2, IFNγ) had lower expression in the sera of OSCC patients [149, 150]. The prior upregulation of GATA-3 in PD patients may augment the GATA-3 suppressive effect on Th1 in OSCC impairing the Th1 mediated immune response.

On the contrary, the Th2 subsets are involved in weaker innate responses and are more prevalent in OSCC facilitating the progression of cancer cells [151]. A very recent study highlighted that, in tongue squamous cell carcinoma, the immune response was biased toward Th2, with their cytokines (IL-4, IL-10) suppressing the cytotoxicity of CTL [152]. The involvement of these Th2 cells has been established in PD; however, in this context it has been suggested that Th2 cells promote a generalized state of immunosuppression through additional means. The elevated levels of the Th2-secreted cytokine, IL-4, within the inflammatory milieu induces CCR4-dependent recruitment of immunosuppressive T-regs into the periodontium [153]. Together, this Th2-mediated suppression of the adaptive immune response in PD is likely to exhibit similar defective mechanisms toward any forthcoming OSCC lesions.

It has only recently been suggested that the Th17 subset might play a role in periodontal diseases. As the principal producers of IL-17, Th17 cells play a central role in recruiting PMNs by upregulating the CXCL8 expression [154]. Th17 cells are now accepted as one of the critical upregulated cellular elements present in periodontally inflamed sites. Indeed, their presence was shown to be directly proportional to probing depths in patients with PD [155], and higher IL-17 levels were detected in the saliva, serum and GCF of diseased patients [155, 156]. Generally, Th17 cells have been linked to unfavorable prognostic outcomes in patients with cancer in the head and neck region [157]. This may come as a result of the tumor promoting activities of IL-17, which regulates the levels of VEGF-A and IL-6 promoting the proliferation of oral cancer cell [157].

## Changes in the Periodontal Microenvironment That Contribute to the Pathogenesis of OSCC

**Figure 3:**
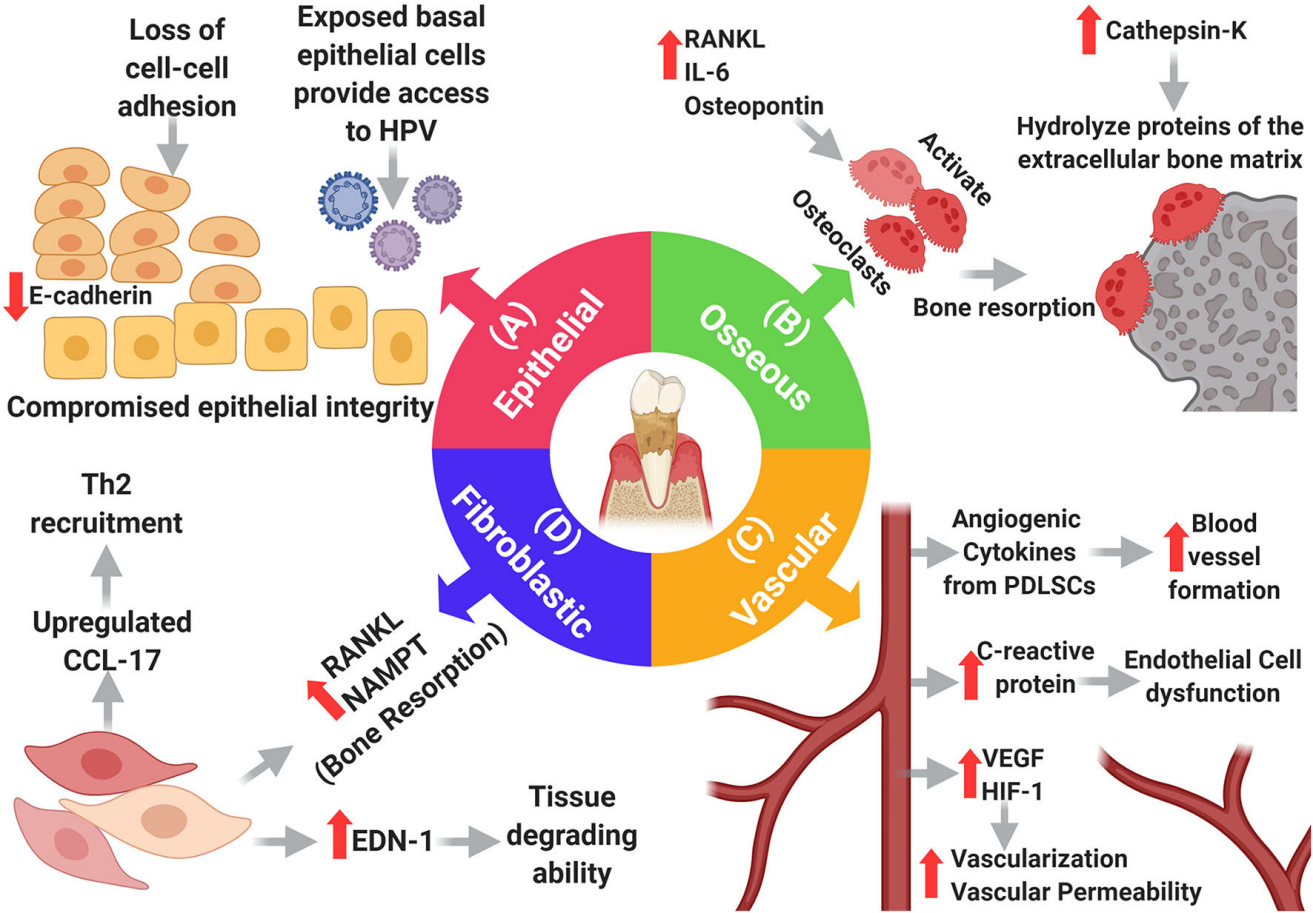
Changes in the main resident cells of the periodontium in periodontitis. **(A)** Epithelial changes: loss of adhesion molecules results in loss of cell-cell adhesion compromising the integrity of the oral epithelium which exposes basal cells providing a portal of entry to HPV. **(B)** Osseous changes: increased RANKL, IL-6, and Osteopontin promote osteoclast-dependent bone resorption, and increased cathepsin-k promotes hydrolysis of extracellular bone proteins. **(D)** Fibroblastic changes: fibroblasts adopt new functions in periodontitis as they upregulate endothelin-1, which in turn increases their tissue degrading ability, moreover, they induce bone resorption by upregulating RANKL and NAMPT. Additionally, fibroblasts upregulate CCL-17, causing expansion of the Th2 cell population, thereby weakening the immune response. **(C)** Vascular changes: many elements within the inflammatory setting induce vascular growth including PDLSCs. VEGF and HIF-1 contribute to angiogenesis, but also, increase the permeability of blood vessels. Increased levels of C-reactive protein cause endothelial cell dysfunction.

The interactions that cancer cells make with their surrounding environment are critical for tumor initiation and progression. Changes in the oral microenvironment in periodontitis can profoundly impact the development of oral cancer. Periodontal inflammation and associated bacterial pathogens cause damage to, and otherwise alter, the specialized tissue that surrounds the teeth (epithelium, connective tissue, vasculature, and bone). These changes can increase the potential for metastatic seeding, survival and growth of cancer cells, explaining why the gingiva, out of all the oral soft tissues, is the most common site for developing metastatic oral carcinomas [23]. Therefore, it is important to discuss some of these PD-induced changes and how they can potentially affect carcinogenesis. Here, we focus on specific changes that may directly influence OSCC [3] (Figure 3).

### Epithelial Changes

The oral epithelial barrier has a critical function of protecting its underlying structures from pathogenic invaders, exogenous substances and mechanical stresses excreted by masticatory forces [158]. Once pathogenic microorganisms breach this protective barrier, gaining access to the tooth supporting structures, they elicit a chronic, persistent inflammatory state with subsequent connective tissue and bone destruction as the severity of inflammation increases [159]. Therefore, disruption of the epithelial barrier and ingress of pathogenic bacteria can cause further epithelial injury in a feed-forward manner. Downregulated expression of E-cadherin, an adhesion molecule that is fundamental for the cell-cell adhesion in the epithelial monolayer, contributes to the compromised integrity of the epithelium. Reduced E-cadherin expression is partly a consequence of increased macrophage production of TNF-α, a well-known E-cadherin down-regulator [160]. Loss of E-cadherin marks the advancement of premalignant, benign lesions into aggressive, metastatic prone carcinomas and is considered a hallmark of EMT in OSCC [161]. Upon exposure to periodontal pathogens, primary oral keratinocytes show increased nuclear activity of Snail-1 [162], which results in down-regulated E-cadherin, and increased expression of vimentin, another EMT biomarker.

The transcription factor, Twist, also an EMT mediator, increases the motility of epithelial cells allowing them to detach and adopt invasive characteristics of mesenchymal-like cells [161]. Higher Twist expression is coupled with higher grades of dysplasia in oral premalignant lesions, making it a useful indicator of malignant transformation in oral epithelium [161]. When periodontal pathogens were co-cultured with OSCC cell lines, Twist expression was upregulated in the malignant cells, and these cells had increased migration [163]. This suggests that PD promotes upregulation of Twist expression, and facilitates cell migration and EMT. The inflamed periodontal tissues also show differential expression of several members of the laminin family including (laminin gamma 2 and laminin beta 3) [164, 165]. These glycoproteins contribute to cancer-related cellular processes, and interact with receptors expressed by cancer cells to stimulate production of collagenase IV, which facilitates cancer cell migration [166]. The same laminin members that were upregulated in PD were also found to be upregulated in OSCC [167].

The loss of cellular adhesion, in addition to the continuous deepening of the periodontal pocket, results in successive exposure of basal epithelial cell layers. As basal cells are the exclusive cellular targets for human papillomavirus (HPV) in the oral cavity, these exposed basal cells provide access points to HPV infections [168]. Further proliferation of these cells favors the persistence of the HPV infection by sustaining viral replication, thus making the infected periodontal pockets reservoirs of this virus [168]. As a central risk factor for cancer development, around 70% of oropharyngeal cancers have been linked to HPV infections [169]. Although only a few studies have explored this association, strong evidence supports the involvement PD mediates HPV-induced carcinogenesis in the oral cavity. Additional longitudinal studies to confirm this interaction will help to reinforce the insight that maintaining periodontal health may prevent the deleterious effects of HPV, specifically in relation to cancer.

### Osseous Changes

When untreated, the breakdown of the periodontal tissue and the subsequent bone destruction in PD ultimately results in tooth loss. This devastating consequence comes as a result of not only microbial invasion, but most importantly, the elicited host inflammatory response, which is central to this local osteolytic process. Hints for an association between osseous changes that occur in PD and the pathogenesis of OSCC have been suggested. The bony changes that occur in PD are involved in the underlying molecular and cellular etiopathological mechanisms governing OSCC, and can also be considered significant risk predictors of OSCC. A study reported that there is a 5.2-fold increase in the risk of developing squamous cell carcinoma of the tongue with every millimeter alveolar bone loss observed in patients with PD [170].

Osteoclast regulators, RANKL, its receptor RANK, and decoy receptor OPG are the core elements orchestrating the osteo-immune bone destruction in PD. Elevated RANKL expression by many inflammatory cell types including, but not limited to, B and T lymphocytes and stromal cells, has been shown at sites with destructive periodontal activity [171]. RANKL induces maturation of osteoclasts, which are directly responsible for resorption of the dento-alveolar bone complex. Treating periodontal conditions was found to result in a significant reduction of RANKL levels in the gingival crevicular fluid, confirming the significance of RANKL in the inflammatory periodontal process [172]. In oral cancer, this amplified osteoclastic activity in PD supports the invasion of cancer cells into bone, which promotes their metastatic spread [173].

Many other osteoclastogenic cytokines are predominant in PD, disrupting the balance between bone resorption and bone formation. IL-6, a cytokine that has been identified as being upregulated in both the saliva and serum in PD, was shown to induce the activation of local osteoclasts and as a result potentiate bone resorption in periodontally inflamed sites [174, 175]. As for its role in oral malignancies, cases of OSCC that harbored greater osteoclast-derived IL-6 mRNA expression were associated, to a larger extent, with mandibular invasion, demonstrating its pro-invasive capacity in OSCC [176]. Osteopontin (OPN), a major non-collagenous bone protein, is key for many biological activities and is well-known for its involvement in bone diseases including PD. OPN enhances the proliferative and differentiation abilities of osteoclasts, and also suppresses osteoblasts [177]. Recently, an important role of OPN was revealed when it was found to induce nuclear translocation and phosphorylation NF-κB, which upregulates the transcription of genes that encode the production of bone damaging products in osteoclasts [177]. This OPN rich environment in PD is likely to also boost osteoclasts-mediated bone resorption in the context of OSCC. Furthermore, OPN was shown to have direct effects on cancer cells. When OSCC cells were cultivated in the presence of OPN, they developed greater adhesive, proliferative, and invasive capacities and expressed greater levels of IL-6 and IL-8 [178].

The cysteine protease Cathepsin K, known to hydrolyze proteins of the extracellular bone matrix, was increased in the GCF of PD patients, with its concentration reflecting disease severity [179]. Mice with experimentally induced PD that were treated with odanacatib, a small molecular inhibitor of Cathepsin K, significantly suppressed the activity of osteoclasts, essentially weakening the bone resorptive process [180]. The involvement of Cathepsin K in PD may contribute to more destructive forms of OSCC, since upregulation of Cathepsin K was shown to be linked with lymph node metastasis and unfavorable prognosis in OSCC [181].

### Fibroblast Changes

Gingival fibroblasts are the most abundant cellular component of the periodontal connective tissue [182]. They function to shape the structural framework of the tissue by continuously regulating the turnover of the extra cellular matrix. However, they also have functions beyond their structural role, and make important contributions to pathological processes. Fibroblasts are one of the first cells that encounter pathogenic threats, and they are able to produce inflammatory mediators, and induce the expression of RANKL and OPG in PD. Invading periodontal micro-organisms were found to upregulate the expression of RANKL and reduce OPG expression in gingival fibroblasts [183]. Pathogen exposed fibroblast exhibit a similar phenotype to those found in the OSCC TME. Cancer associated fibroblasts (CAF) have also been observed to modulate RANKL expression, contributing to bone invasion by promoting osteoclastogenesis [184].

Several other key GF products have been shown to have altered expression upon encountering pathogenic bacteria, and these changes mirror the CAF phenotype present in the TME of OSCC. *P. gingivalis* prompts the expression of Galectin-1 (Gal-1), a beta-galactoside-binding protein, in gingival fibroblasts [185]. This deregulates the cytokine network in PD, limiting the migration of leukocytes and thereby allowing further colonization by pathogenic bacteria. In OSCC, Gal-1 was found to activate CAF and their expression of alpha-smooth muscle actin (α-SMA) inducing their transdifferentiation into myofibroblasts [186]. This results in upregulated expression of fibroblast monocyte chemotactic protein-1 (MCP-1), which then binds to CCL2 on cancer cells, promoting the metastatic ability of these malignant cells. Knockdown of Gal-1 in CAF results in quiescence of these fibroblasts and reverses their ability to promote migration of cancer cells [186].

Other molecules that govern the expression of different GF products show comparable contributions in both PD and OSCC [187, 188]. For instance, the pleiotropic peptide endothelin-1 (EDN-1) is elevated in both diseases. EDN-1 induces fibroblast tissue degrading capacity and breakdown of the oral stroma, which can promote the invasion by OSCC cells [188]. Nicotinamide phosphoribosyltransferase (NAMPT) regulates GFs in both diseases [189, 190]. In PD, NAMPT is critical for inducing GF expression of bone destructive mediators, including COX2, MMP1, and MMP3, supporting progressive bone loss [189]. In OSCC, upregulation of NAMPT was observed, suggesting it may play a role in oral dysplastic pathologies [190].

### Vascular Changes

Aberrant vascularization is one of the most prominent features of inflamed periodontium. Many cells contribute to the vascularization process, which is intensified by the dense inflammatory infiltrate. Periodontal ligament stem cells (PDLSCs) are one of the most prominent cell types involved in this process. In the inflammatory milieu, PDLSCs promote angiogenesis by activating autophagy, which in turn enhanced the production of angiogenic cytokines including basic fibroblast growth factor (bFGF) and angiogenin [191]. Although vascular density was found to be increased in PD, endothelial and microvascular dysfunction has been reported in cases of severe PD [192]. Moreover, elevated levels of IL-6 and C-reactive protein (CRP) in the peripheral blood of periodontal patients was found to predispose to an increased risk of developing peripheral arterial diseases which are regarded as an independent risk factors for developing cancer [193, 194].

CRP, a plasma protein mainly produced by the liver, is a valuable inflammatory biomarker and has been shown to promote endothelial dysfunction. In PD, levels of this protein were found to be elevated both in the local inflammatory site as well as systemically [193]. It has been recognized that this local increase comes as a result of stimulation of endothelial cells by IL-6 and IL-1β produced by other inflammatory cells. This local increase was augmented by extra-hepatic production of CRP to induce the systemic elevation manifested in PD. High CRP levels are associated with OSCC patients that had metastatic lesions, suggesting that it could be used as a predictor of lymph node metastasis [195]. In addition, it has been suggested that a higher CRP/albumin ratio might be an independent prognostic marker of poor outcomes in patients with OSCC [196]. Since CRP affects the metastatic ability of cancer cells this could represent another potential avenue whereby PD interacts with OSCC.

The expression of hypoxia inducible factor-1 (HIF-1) and vascular endothelial growth factor (VEGF) in periodontal patients was shown to be greater than that of healthy individuals [197]. VEGF, a fundamental growth factor that stimulates the proliferation of endothelial cells and increases vascular permeability, is regulated by HIF-1. The expression of both cytokines was found to correlate with the severity of the periodontal condition [197]. HIF-1α also provokes invasion of OSCC cells, and VEGF-dependent angiogenesis was found to be vital for continuous vascular expansion during the transition from epithelial dysplasia to carcinoma [198, 199].

## Conclusion

In an era where understanding the putative interconnectivity between cancer and other diseases, particularly inflammatory diseases, unveils new aspects and targets for cancer therapy, it has become clear that PD has the potential to exacerbate all the pathogenic characteristics of OSCC. From the earliest steps of the neoplastic process, PD may, in part, contribute to the induction of permanent genomic alterations, due to the sustained presence of bacterial-derived mutagens. Furthermore, primary and metastatic tumor cells can benefit from the weakened stromal elements due to PD-dependent tissue destruction, and their proliferation in this environment is sustained by its rich growth/survival factors. In addition to potentially mutagenic properties and stromal defects, the oral inflammatory environment in PD is permissive for tumor progression. This is characterized by increased inflammatory infiltrate, which is nevertheless associated with a blunted or suppressed immune response that contributes to the development and progression of OSCCs. PD can therefore aid the development of oral cancers that would otherwise be destroyed in a healthy oral environment by normal immune cell functions. Therefore, monitoring oral health and providing effective treatments for PD might hold promise in normalizing this misdirected immune response, restoring an inflammatory network capable of exerting anti-tumor properties.

## Author Contributions

OE designed and drafted the manuscript. AB, NF, and HT reviewed and edited the manuscript. MG organized, provided the frame for the manuscript, and critically revised the content. All authors contributed to the article and approved the submitted version.

## Conflict of Interest

The authors declare that the research was conducted in the absence of any commercial or financial relationships that could be construed as a potential conflict of interest.
